# Myocarditis and coronavirus disease 2019 vaccination: A systematic review and meta-summary of cases

**DOI:** 10.17305/bb.2022.8779

**Published:** 2023-08-01

**Authors:** Pandit Bagus Tri Saputra, Roy Bagus Kurniawan, Desy Trilistyoati, Makhyan Jibril Al Farabi, Hendri Susilo, Mochamad Yusuf Alsagaff, Yudi Her Oktaviono, Henry Sutanto, Arief Gusnanto, Citrawati Dyah Kencono Wungu

**Affiliations:** 1 Department of Cardiology and Vascular Medicine, Faculty of Medicine, Universitas Airlangga–Dr Soetomo General Academic Hospital, Surabaya, Indonesia; 2 Faculty of Medicine, Universitas Airlangga, Surabaya, Indonesia; 3 Department of Cardiology and Vascular Medicine, Universitas Airlangga Hospital, Surabaya, Indonesia; 4 Department of Cardiology, CARIM School for Cardiovascular Diseases, Maastricht University, Maastricht, The Netherlands; 5 School of Mathematics, University of Leeds, Leeds, UK; 6 Department of Physiology and Medical Biochemistry, Faculty of Medicine, Universitas Airlangga, Surabaya, Indonesia; 7 Institute of Tropical Disease, Universitas Airlangga, Surabaya, Indonesia

**Keywords:** Myocarditis, coronavirus disease 2019 (COVID-19) vaccination, vaccine, severe acute respiratory syndrome coronavirus 2 (SARS-CoV-2), side effect

## Abstract

Vaccination is significant to control, mitigate, and recover from the destructive effects of coronavirus disease 2019 (COVID-19). The incidence of myocarditis following COVID-19 vaccination has been an increasing and growing public concern; however, little is known about it. This study aimed to systematically review myocarditis following COVID-19 vaccination. We included studies containing individual patient data of myocarditis following COVID-19 vaccination published between January 1, 2020 and September 7, 2022 and excluded review articles. Joanna Briggs Institute critical appraisals were used for risk of bias assessment. Descriptive and analytic statistics were performed. A total of 121 reports and 43 case series from five databases were included. We identified 396 published cases of myocarditis and observed that the majority of cases were male patients, happened following the second dose of mRNA vaccine administration, and experienced chest pain as a symptom. Previous COVID-19 infection was significantly associated with the risk of myocarditis following the administration of the first dose (*p* < 0.01; OR, 5.74; 95% CI, 2.42–13.64), indicating that its primary mechanism is immune mediated. Moreover, 63 histopathology examinations were dominated by non-infective subtypes. Electrocardiography and cardiac marker combination is a sensitive screening modality. However, cardiac magnetic resonance is a significant non-invasive examination to confirm myocarditis. Endomyocardial biopsy may be considered in confusing and severe cases. Myocarditis following COVID-19 vaccination is relatively benign, with a median length of hospitalization of 5 days, intensive care unit admission of <12%, and mortality of <2%. The majority was treated with non-steroidal anti-inflammatory drugs, colchicine, and steroids. Deceased cases had characteristics of being female, older age, non-chest pain symptoms, first-dose vaccination, left ventricular ejection fraction of <30%, fulminant myocarditis, and eosinophil infiltrate histopathology. Systematic review registration: PROSPERO (CRD42021271806).

## Introduction

Since first identified and reported in China in late 2019, coronavirus disease 2019 (COVID-19) has rapidly and extensively spread among countries, escalating into a pandemic. The COVID-19 pandemic has severely affected economic, social, political, and cultural sectors [1] and has persisted in attracting worldwide attention.

To control, mitigate, and recover from the destructive effects of COVID-19, vaccination is critical. Recent evidence regarding the ineffectiveness of COVID-19 vaccines for some strains. However, COVID-19 vaccination may pose beneficial effects, particularly, for high-risk populations, including older adults and individuals with multiple comorbidities [2]. To support international vaccination coverage, the World Health Organization (WHO) issued authorization for COVID-19 vaccine emergency use [3] in combination with government regulation to accelerate [4] COVID-19 vaccination up to 12 billion doses, which continues to increase globally [5]. With the rapid increase in vaccination shots comes several reported adverse events complained by patients, ranging from minor to major events [6].

COVID-19 vaccination induced several adverse symptoms including pain at the injection site, fever, myalgia, arthralgia, headache, and abdominal discomfort [6, 7]. Several vaccine-related diseases have also been reported in different organs, including the respiratory tract (e.g., asthma attack, diffuse alveolar hemorrhage, and eosinophilic pneumonia), gastrointestinal tract (e.g., appendicitis, autoimmune hepatitis, intestinal ulcer, and perforation), skin (e.g., alopecia areata, bullous pemphigoid, psoriasis, and eosinophilic cellulitis), and endocrine organs (e.g., Graves’ disease, hypophysitis, hypothyroidism, and diabetes mellitus) [8]. Emerging myocarditis cases have received media attention. Various literature reported myocarditis following COVID-19 vaccination, which is considered one of the serious adverse events [9]. The number of these reports has been increasing as more individuals get vaccinated, thereby growing public concern regarding COVID-19 vaccination [10].

Therefore, to provide appropriate information and a standing position for the public and clinicians, including myocarditis following COVID-19 vaccination, any vaccination-related issues should be thoroughly investigated. However, most reports on myocarditis following COVID-19 vaccination were case reports and case series, and little is known regarding the nature and characteristics of this phenomenon. Therefore, this systematic review aimed to describe the characteristics of myocarditis following COVID-19 vaccination by collecting detailed individual patient data (IPD) from those available reports and literature to create a systematic review and meta-summary of cases of myocarditis following COVID-19 vaccination.

## Materials and methods

This review followed the Preferred Reporting Items for Systematic Reviews and Meta-Analyses (PRISMA) 2020 [11] and was registered and can be accessed in the PROSPERO database (CRD42021271806). No ethical approval was required as we only analyzed secondary data from published literature.

### Eligibility criteria

The following study types were included in this review: case reports, case series, and observational studies (cross-sectional, case-control, and cohort studies). Screening of eligible studies was based on the following inclusion criteria: (1) patients of all ages with myocarditis following any COVID-19 vaccination type as participants according to the definition from European Society of Cardiology (ESC) guidelines for myocarditis [12] or Centers for Disease Control and Prevention (CDC) updates on myocarditis following COVID-19 vaccination [13]; (2) individual-specific patient data were available; (3) all COVID-19 vaccine types (mRNA and non-mRNA vaccines); and (4) reported in English. Diagnosis of myocarditis was based on the combination of clinical signs, electrocardiography (ECG), cardiac markers, echocardiography/angiocardiography, cardiac magnetic resonance (CMR), and, if available, histopathologic examination (autopsy or endomyocardial biopsy) according to the ESC [12] and CDC updates on myocarditis [13].

We excluded nonscientific articles (popular magazines), randomized-controlled trials, reviews, unavailable text, duplication, and studies that report unclear populations (e.g., pericarditis without myocardial involvement or heart failure and myocardial injury instead of myocarditis). Myocarditis that was not due to COVID-19 was excluded. If needed, we contacted the authors to ask for additional patient information.

### Search strategy

On September 7, 2022, a literature search was systematically performed on ScienceDirect, Scopus, PubMed, CINAHL via EBSCO, and ProQuest using keywords and medical subject headings with English language restrictions. Published articles from January 1, 2020, the month of COVID-19 discovery, to September 7, 2022 were included. The search terms were (“COVID19” OR “COVID-19” OR “coronavirus disease 2019” OR “SARS-CoV-2”) AND (“vaccine” OR “vaccination”) AND (“myocarditis” OR “myocardial injury” OR “myopericarditis”).

### Study selection

Titles and abstracts were independently screened for eligibility. Subsequently, studies with potentially eligible abstracts were reviewed for full-text articles according to previously determined inclusion and exclusion criteria. Any disagreements were resolved by consensus of those authors.

### Data extraction

The following data were extracted from included studies: the number of cases; sex; age; region; comorbidities; previous COVID-19 infection; current COVID-19 infection status; vaccine types; vaccine doses; from the day(s) of vaccination to specific myocarditis symptoms; from the day(s) of vaccination to the emergency department (ED) admission; from the day(s) of specific symptom onset to ED from specific myocarditis symptoms; preceding symptoms; presenting symptoms; cardiac markers, including troponin I, high-sensitive troponin I (hsTnI), troponin T, high-sensitive troponin T (hsTnT), creatinine phosphokinase (CPK), creatinine kinase myocardial band (CKMB), brain natriuretic peptide (BNP), and Pro-BNP; D-dimer; white blood cells (WBCs); erythrocyte sedimentation rate (ESR); C-reactive protein (CRP); ECG/Holter stress test; echocardiography/angiography; CMR; cardiac histopathologic examination; length of hospitalization (LoH); management; and outcomes (deceased or recovery).

In the context of day(s) from the event (e.g., vaccination day) to the event (e.g., specific symptoms), the first event was determined as day one. The specific symptoms included chest pain, dyspnea, palpitation, syncope, or cardiac arrest [13], which led patients to the ED. Preceding symptoms were determined as any symptoms or signs that occurred before specific symptoms. CMR data were collected and mainly classified as edema, hyperemia, fibrosis/scar, and pericardial involvement [12], [14]. Patients were classified into probable and confirmed cases according to the CDC criteria [13], and their myocarditis score was also evaluated based on the ESC criteria [12]. All collected data were independently compared as pre-piloted forms, and any discrepancy was resolved through consensus.

### Quality assessment

We independently analyzed and judged the risk of bias of included studies using the Joanna Briggs Institute (JBI) for case reports and case series critical appraisal instruments [15]. The JBI critical appraisal for case reports consists of eight questions, whereas JBI critical appraisal for case series consists of ten questions. However, we combined questions number 4 and 5 in the JBI appraisal as they represent the same domain (bias selection of participants into the study), and we did not include question number 10, considering statistical analysis is not an appropriate characteristic in this context.

**Figure 1.**
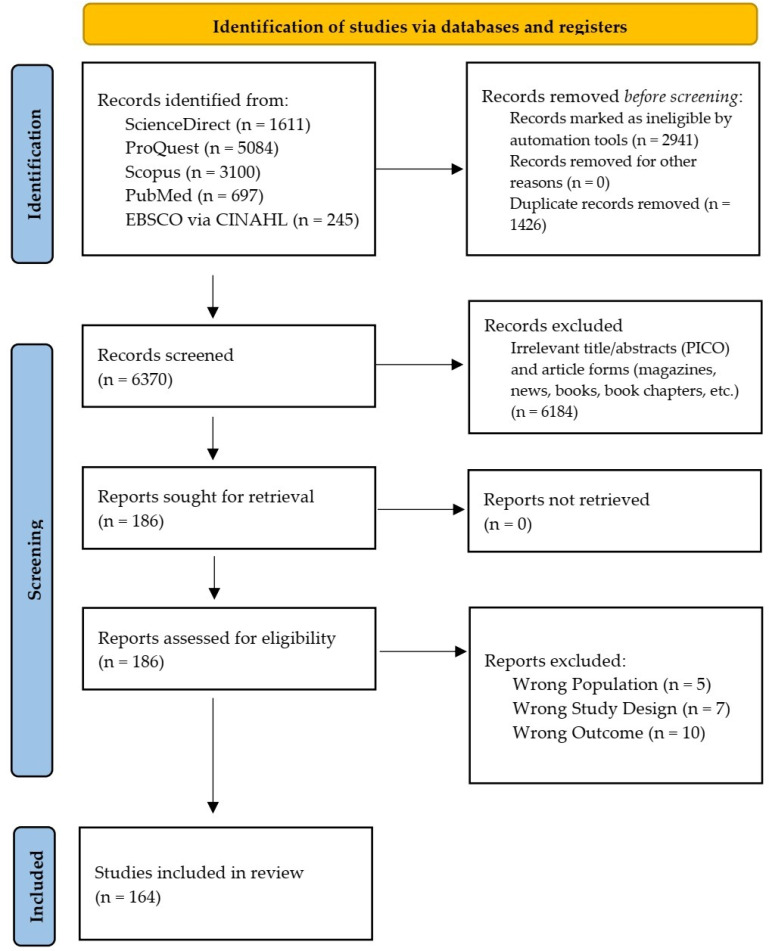
Preferred reporting items for systematic reviews and meta-analyses flow diagram of the study selection process.

This critical appraisal consists of yes/no/unclear answers for each question [16]. The studies were classified as low, medium, and high risks of bias according to the total “yes” answers of ≥74%, 50%–74%, and ≤49% of total questions, respectively.

### Statistical analysis

All collected IPD from the included study were combined in a meta-summary. Patients with confirmed cases were compared with probable cases. A *p*-value threshold of 0.05 was considered statistically significant. The Shapiro–Wilk test was used to determine the normality distribution of the data [17]. Categorical variables were presented as frequencies and percentages. Continuous variables were presented as means and standard deviations; otherwise, median and range (minimum–maximum) were considered. As indicated, categorical data were compared using Chi-square or Fisher’s exact test [18]. Moreover, normally distributed data were compared by employing the independent T-test. Otherwise, the Mann–Whitney test would be used when the data were abnormally distributed by the Shapiro–Wilk test. We identified demographic and comorbid factors that may contribute to the occurrence of myocarditis following the first COVID-19 vaccination by multivariate analysis (logistic regression). Only variables with a *p*-value of <0.25 were included in the multivariate analysis [19]. The Spearman or Pearson correlation test was used to evaluate the correlation between clinical parameters and outcomes by considering data distribution. We used a statistical software, IBM SPSS Statistics for Windows, version 22 (IBM Corp., Armonk, NY, USA), during statistical analysis processes.

## Results

### Study selection

In the primary search, 6370 studies were identified after the removal of 2941 irrelevant research articles by the automation feature of each search engine and 1426 duplications. A total of 6184 articles were excluded during the title and abstract screening, leaving 186 studies for full-text reviews. Subsequently, 24 studies were excluded, while 164 studies consisted of 121 case reports [20–130] and 43 case series [131–178], with a total of 396 cases included (Figure 1) in this meta-summary. Some studies, such as those by Diaz et al. [179] and Montgomery et al. [180] fulfilled the inclusion criteria and consisted of several participants. However, we excluded them because specific individual data could not be extracted. We only included patients who fulfilled the myocarditis criteria, such as in the case series reported by Patel et al. [178].

### Meta-summary of cases

IPD from 396 cases was included in this systematic review (Table S2). The basic data and comparison of demographic, clinical characteristics, and management between probable and confirmed cases are presented in Table 1.

**Table 1:** Characteristics and comparisons between published probable and confirmed cases of myocarditis following vaccination

			**CDC VAERS Myocarditis Status^#^ [13]**		
**Variables**	Reported cases*	Percentage or median (%)**	**Probable (%)**	**Confirmed (%)**	***p*-value^a^**
**Age**	370	22 (12–88)^b^	20 (12–88)^b^	23 (12–80)^b^	0.04^c^
**Male**	384	361 (87.6)	73 (90.1)	263 (86.8)	0.57^h^
**WHO regional area	383				
Africa (AFR)		1 (0.3)	1 (1.2)	0	N/A
America (AMR)		150 (39.2)	33 (40.2)	117 (38.9)	N/A
South-East Asia (SEAR)		2 (0.5)	1 (1.2)	1 (0.3)	N/A
Europe (EUR)		155 (40.5)	27 (32.9)	128 (42.5)	N/A
Eastern Mediterranean (EMR)		16 (4.2)	10 (12.2)	6 (2.0)	N/A
Western Pacific (WPR)		59 (15.4)	10 (12.2)	49 (16.3)	N/A
**Vaccine type	394				
BNT16 2b2		270 (68.5)	61 (75.3)	209 (66.8)	N/A
mRNA-1273		97 (24.6)	16 (19.8)	81 (25.9)	N/A
Ad26.COV2. S		8 (2.0)	2 (2.5)	6 (1.9)	N/A
AZD1222		14 (3.6)	2 (2.5)	12 (3.8)	N/A
BBV152		1 (0.3)	0 (0)	1 (0.3)	N/A
rAd26 and rAd5		1 (0.3)	0 (0)	1 (0.3)	N/A
NVX-C oV2373		1 (0.3)	0 (0)	1 (0.3)	N/A
Inactivated SARS-CoV-2		2 (0.5)	0 (0)	2 (0.6)	N/A
**Previous SARS-CoV-2 infection	167	33 (19.8)	4 (11.1)	29 (22.1)	0.22^h^
**COVID-19 vaccination history**	384				
First dose		89 (23.2)	16 (19.8)	73 (24.1)	0.50^h^
Second/Third doses		295 (76.8)	65 (80.2)	230 (75.2)	
**Comorbidities**	231				
None		131 (56.7)	24 (42.9)	107 (61.1)	0.03^g,h^
Psychiatric/functional disorders		14 (6.1)	4 (7.1)	10 (5.7)	0.75
Immune system/inflammatory disorders		14 (6.1)	6 (10.7)	8 (4.6)	0.11
Hypertension		13 (5.6)	5 (8.9)	8 (4.6)	0.31
Bronchial asthma		13 (5.6)	6 (10.7)	7 (4.0)	0.09
Myocarditis		11 (4.8)	5 (8.9)	6 (3.4)	0.14
Dyslipidemia		10 (4.3)	3 (5.4)	7 (4.0)	0.71
Miscellaneous atopic disease		6 (2.6)	1 (1.8)	5 (2.9)	1.00
GERD/gastritis		5 (2.2)	1 (1.8)	4 (2.3)	1.00
Dysrhytmia		5 (2.2)	1 (1.8)	4 (2.3)	1.00
Hypothyroidism		5 (2.2)	1 (1.8)	4 (2.3)	1.00
Genetic/chromosomal disease		5 (2.2)	2 (3.6)	3 (1.7)	0.60
Obesity		4 (1.7)	3 (5.4)	1 (0.6)	0.05^g^
DMT2/insulin resistance		4 (1.7)	3 (5.4)	1 (0.6)	0.05^g^
Coronary arterial disease		4 (1.7)	3 (5.4)	1 (0.6)	0.05^g^
Malignancy		4 (1.7)	0	4 (2.3)	0.58
Infections		3 (1.3)	0	3 (1.7)	1.00
Congestive heart failure		2 (0.9)	2 (3.6)	0	0.06
Multiple sclerosis		2 (0.9)	2 (3.6)	0	0.06
Obstructive sleep apnea		1 (0.4)	0	1 (0.6)	1.00
Aortic root dilatation		1 (0.4)	0	1 (0.6)	1.00
Vitiligo		1 (0.4)	1 (1.8)	0	0.24
COPD		1 (0.4)	1 (1.8)	0	0.24
Recurrent pneumothorax		1 (0.4)	0	1 (0.6)	1.00
Encephalopathy		1 (0.4)	1 (1.8)	0	0.24
**Preceding symptoms**	215				
None		71 (33.0)	20 (43.5)	51 (30.2)	0.13^h^
Fever		96 (44.7)	15 (32.6)	81 (47.9)	0.09^h^
Myalgia		47 (21.9)	8 (17.4)	39 (23.1)	0.53^h^
Malaise		31 (14.4)	5 (10.9)	26 (15.4)	0.60
Headache		24 (11.2)	5 (10.9)	19 (11.2)	1.00^h^
Chills		21 (9.8)	5 (10.9)	16 (9.5)	0.78
Nausea		10 (4.7)	2 (4.3)	8 (4.7)	1.00
Diarrhea		10 (4.7)	2 (4.3)	8 (4.7)	1.00
Vomiting		9 (4.2)	4 (8.7)	5 (3.0)	0.10
Dry cough		9 (4.2)	1 (2.2)	8 (4.7)	0.69
Palpitation		9 (4.2)	0	9 (5.3)	0.21
Shortness of breath		9 (4.2)	3 (6.5)	6 (3.6)	0.41
Limb pain		7 (3.3)	1 (2.2)	6 (3.6)	1.00
Diaphoresis		5 (2.3)	1 (2.2)	4 (2.4)	1.00
Injection site pain		4 (1.9)	0	4 (2.4)	0.58
Arthralgia		4 (1.9)	0	4 (2.4)	0.58
Sore throat		4 (1.9)	0	4 (2.4)	0.58
Back pain		3 (1.4)	1 (2.2)	2 (1.2)	0.52
Abdominal pain		2 (0.9)	1 (2.2)	1 (0.6)	1.00
Hematuria		1 (0.5)	0	1 (0.6)	1.00
Epistaxis		1 (0.5)	0	1 (0.6)	1.00
**Presenting symptoms**	387				
Chest pain		323 (83.5)	68 (84.0)	255 (83.3)	1.00^h^
Fever		57 (14.7)	9 (11.1)	48 (15.7)	0.39^h^
Dyspnea or SoB		56 (14.5)	12 (14.8)	44 (14.4)	1.00^h^
Malaise/fatigue		35 (9.0)	11 (13.6)	24 (7.8)	0.17^h^
Headache		25 (6.5)	5 (6.2)	20 (6.5)	1.00^h^
Tachycardia/palpitation		23 (5.9)	6 (7.4)	17 (5.6)	0.60
Gastrointestinal symptoms		18 (4.7)	4 (4.9)	14 (4.6)	1.00
Myalgia		15 (3.9)	2 (2.5)	13 (4.2)	0.75
Chills		10 (2.6)	3 (3.7)	7 (2.3)	0.44
Diaphoresis		7 (1.8)	1 (1.2)	6 (2.0)	1.00
Syncope		7 (1.8)	1 (1.2)	6 (2.0)	1.00
Hypotension		6 (1.6)	1 (1.2)	5 (1.6)	1.00
Limb pain		4 (1.0)	1 (1.2)	3 (1.0)	1.00
Shock		4 (1.0)	1 (1.2)	3 (1.0)	1.00
Arrest		3 (0.8)	0	3 (1.0)	1.00
Athralgia		1 (0.3)	0	1 (0.3)	1.00
Paresthesia		1 (0.3)	1 (1.2)	0	0.21
Heart failure		1 (0.3)	0	1 (0.3)	1.00
Cough		1 (0.3)	1 (1.2)	0	0.21
Back pain		1 (0.3)	0	1 (0.3)	1.00
**From vaccination to specific symptoms (days)**	344	3 (1–90)^b^	3 (1–60)^b^	3 (1–90)^b^	0.00^c,g^
**From vaccination to the ED (days)**	211	4 (1–90)^b^	3 (2–60)^b^	4 (1–90)^b^	0.28^c^
**Specific symptoms to the ED (days)**	202	0 (0–21)^b^	0 (0–18)^b^	0 (0–21)^b^	0.03^c,g^
**Clinical laboratory^f^**					
CRP	262	205 (78.2)	36 (81.8)	169 (77.5)	0.68^h^
White blood cells	110	44 (40.0)	10 (45.5)	34 (38.6)	0.73^h^
ESR	67	35 (52.2)	10 (66.7)	25 (48.1)	0.33^h^
D-dimer	44	24 (54.5)	11 (68.8)	13 (46.4)	0.26^h^
**Cardiac-specific examination^f^					
hsTroponin I	48	43	3 (100.0)	40 (88.9)	1.00
Troponin I	177	165 (93.2)	47 (97.9)	118 (91.5)	0.18
hsTroponin T	43	42 (97.7)	3 (75.0)	39 (100.0)	0.09
Troponin T	74	67 (90.5)	9 (81.8)	58 (92.1)	0.28
CPK	68	58 (85.3)	10 (90.9)	48 (84.2)	1.00
CKMB	57	48 (84.2)	9 (81.8)	39 (84.8)	1.00
BNP	82	42 (51.2)	5 (38.5)	37 (53.6)	0.48^h^
NT-proBNP	65	47 (72.3)	4 (50.0)	43 (75.4)	0.20
**Left ventricular rejection fraction (LVEF) (%)**	265	55 (5.0–72.0)	57 (10–70)	54 (5.0–72.0)	0.11^c^
LVEF < 50.00 %	265	86 (32.7)	12 (26.7)	74 (33.9)	0.44^h^
**ESC score**	396	3 (1–5)	2 (1–4)^b^	3 (1–5)^b^	<0.01^c,g^
**Length of hospitalization (day)**	248	5 (0–79)	3 (1–16)^b^	5 (0–79)^b^	<0.01^c,g^
**Management**	322				
None/observation		33 (10.2)	9 (11.8)	24 (9.8)	0.76^h^
NSAIDs		177 (55.0)	50 (65.8)	127 (51.6)	0.04^g,h^
Colchicine		81 (25.2)	19 (25.0)	62 (25.2)	1.00^h^
Corticostesroids		64 (19.9)	11 (14.5)	53 (21.5)	0.24 ^h^
Beta-blockers		54 (16.8)	8 (10.5)	46 (18.7)	0.14^h^
ACEIs/ARBs		50 (15.5)	6 (7.9)	44 (17.9)	0.55^h^
Intensive care unit		39 (12.1)	11 (14.5)	28 (11.4)	0.60^h^
IVIGs		34 (10.6)	5 (6.6)	29 (11.8)	0.28^h^
Diuretics		19 (5.9)	2 (2.6)	17 (6.9)	0.26
Inotropic agents		15 (4.7)	3 (3.9)	12 (4.9)	1.00
Gastric acid suppressors		14 (4.3)	6 (7.9)	8 (3.3)	0.11
Antibiotics		11 (3.4)	3 (3.9)	8 (3.3)	0.73
Anticoagulants		10 (3.1)	0	10 (4.1)	0.13
ECMO		10 (3.1)	1 (1.3)	9 (3.7)	0.46
MHS		10 (3.1)	2 (2.6)	8 (3.3)	1.00
Paracetamol		9 (2.8)	4 (5.3)	5 (2.0)	0.22
Antiplatelets		9 (2.8)	1 (1.3)	8 (3.3)	0.69
Miscellaneous analgesia		8 (2.5)	2 (2.6)	6 (2.4)	1.00
Anti-arrhythmias		7 (2.2)	1 (1.3)	6 (2.4)	1.00
Vasodilators		6 (1.9)	2 (2.6)	4 (1.6)	0.62
Intubation/ventilator		5 (1.6)	2 (2.6)	3 (1.2)	0.34
Hemodialysis		4 (1.2)	2 (2.6)	2 (0.8)	0.24
CPR		4 (1.2)	1 (1.3)	3 (1.2)	1.00
Heart failure		2 (0.6)	0	2 (0.8)	1.00
Statin		2 (0.6)	1 (1.3)	1 (0.4)	0.42
Immunosuppressant		2 (0.6)	0	2 (0.8)	1.00
Bronchodilators		1 (0.3)	1 (1.3)	0	0.24
Interleukin antagonist		1 (0.3)	0	1 (0.4)	1.00
MgSO4		1 (0.3)	1 (1.3)	0	0.24
Vitamin D		1 (0.3)	0	1 (0.4)	1.00

^#^The definition of probable and confirmed cases is based on the Centers for Disease Control and Prevention classification of myocarditis [13]. *A case is defined as individual patient data (IPD). Reported cases refer to the number of cases that reported a specific variable. Notably, not all cases describe all variables and that made each variable have a different number of reported cases. For example, reported data on age, sex, and previous COVID-19 infection are available in 370, 384, and 167 cases, respectively. The numbers of reported cases (which act as the denominator for each group) between the probable and confirmed groups are also different (not shown). **This refers to the percentage of variable (with the reported cases of variable as the denominator). Furthermore, this refers to the median in variables with continuous data (e.g., age, from vaccination to the specific symptom, from vaccination to the ED, from specific symptoms to the ED, ESC score, and length of hospitalization). ^a^The *p*-values (rightmost column) of the result of testing the hypothesis between the probable and confirmed groups a Fisher’s exact test; ^b^median (minimum–maximum); ^c^Mann–Whitney test; ^d^mean ± standard deviation; ^e^independent T-test; ^f^an increase of more than their reference ranges; ^g^significant at p ≤ 0.05; ^h^Chi-square test. N/A: Not applicable; ED: Emergency department; DMT2: Diabetes mellitus type 2; GERD: Gastroesophageal reflux disease; COPD: Chronic obstructive pulmonary disease; WBC: White blood cell; ESR: Erythrocyte sedimentation rate; CRP: C-reactive protein; CKMB: Creatinine kinase myocardial band; BNP: B-type natriuretic peptide; NT-proBNP: N-Terminal pro-BNP; ESC: European Society of Cardiology; NSAIDs: Nonsteroidal anti-inflammatory drugs; IVIG: Intravenous immunoglobulin; ACE: Angiotensin-converting enzyme; ECMO: Extracorporeal membrane oxygenation; MHS: Mechanical hemodynamic support; CPR: Cardiopulmonary resuscitation.

Notably, not all cases describe all variables and that made each variable have a different number of reported cases. For example, reported data on age, sex, and previous COVID-19 infection are available in 370, 384, and 167 cases, respectively. That also applies to other variables, which are subsequently named as reported cases in Table 1. Furthermore, due to different reported cases in each variable, the numbers of probable and confirmed cases (which act as the denominator for each group) are different in each variable. The number of probable and confirmed cases for each variable is not shown.

### Demographic characteristics

The majority of reported myocarditis was mRNA-type vaccines, including BNT162b2 (68.5%) and mRNA-1273 (23.9%). Moreover, we observed myocarditis following the administration of viral vector-based vaccine (6%, consisting of 8, 14, and 1 cases of Ad26.COV2.S, AZD1222, and rAd26-rAd5, respectively), inactivated virus vaccine (3 cases), and recombinant protein vaccine (1 case of NVX-CoV2373). The median age was 22 (range 12–88) years, and the majority (87.6%) were male patients. There were 121 cases of children under 18 years old, with eight cases of children under 13 years old. According to WHO regional area, the majority of reported articles were from Europe (40.5), America (39.2%), and Western Pacific (15.2%). Less than 10% of reports were from Asian and African regions.

### Clinical characteristics

Of 231 reported cases, 43.3% had comorbidities, including psychiatric disorders (6.1%), immune/inflammatory disorders (6.1%), hypertension (5.6%), bronchial asthma (5.6%), history of myocarditis (4.8%), dyslipidemia (4.3%), and others (Table 1). There were 33 of 167 (19.8%) reported cases with previous COVID-19 infection. The majority (76.8%) of the cases were associated with the second dose of the COVID-19 mRNA vaccine.

Preceding symptoms accompanied 33% of cases; the most reported preceding symptoms were fever (44.7%), myalgia (21.9%), malaise (14.4%), headache (11.2%), and chills (9.8%). Other preceding symptoms are listed in Table 1.

Chest pain was the most common presenting (83.5%) (Table 1) and myocarditis-specific (97%) symptom (Table S3). Other specific symptoms included dyspnea (14.5%), palpitation (5.9%), hypotension/syncope/heart failure (4.4%), and diaphoresis (1.8%). Three patients (0.8%) had cardiac arrest events in the ED. The three most common constitutional symptoms were fever (14.7%), malaise (9%), and headache (6.5%).

The median of vaccination day to the presence of specific symptoms and from the vaccination day to the ED admission was 3 (1–90) and 4 (1–90) days, respectively. Meanwhile, most cases reported that the patients came to the ED on the day of specific symptom onset. The confirmed group had a significantly shorter interval of the day(s) of vaccination to specific symptoms and day(s) of specific symptoms to the ED (*p* < 0.01 and *p* < 0.03, respectively) than the probable group (Table 1).

### Laboratory examination

In this meta-summary, all inflammatory markers, except for WBC (40%), were increased in more than half of the cases: CRP (78.2%), D-Dimer (54.5%), and ESR (52.2%). No differences in inflammatory marker abnormality between the probable and confirmed groups were noted (Table 1).

### Electrocardiography

Of 352 ECG results, typical abnormalities were noted in 290 (82.3%) cases (Table 2). ST-segment abnormalities were observed as 83% of abnormalities and 68.5% of overall ECG results. The most common ST-segment abnormality was ST-segment elevation (81.7%), whereas 36% was diffuse ST-segment elevation. T-wave abnormalities were noted in 10% of the ECG results. Intraventricular conduction abnormalities (LBBB, RBBB, and nonspecific wide QRS complex) were more common than atrioventricular block (grades I–III) (6.8% vs 1.4%, respectively). Additionally, ventricular-origin arrhythmia was more common than atrial-origin arrhythmia (6.3% vs 1.7%, respectively).

**Table 2:** Cardiac-specific examination findings

**Examination**	**Number of cases (%)**
**ECG**	
*Available ECG^a^*	352 (96)
PR-segment depression	26 (7.4)
ST-segment abnormality	241 (68.5)
ST depression	11 (3.1)
ST elevation*	197 (56)
Nonspecific ST abnormality	29 (8.2)
T abnormality	35 (9.9)
Peak T wave	5 (1.4)
T inversion	24 (6.8)
Unspecified abnormality	16 (4.5)
Intraventricular conduction	24 (6.8)
Left-BBB	3 (0.9)
Right-BBB	14 (4)
Unpacified wide QRS complex (≥0.12 s)	7 (2)
Atrioventricular block (grades I–III)	5 (1.4)
Premature atrial contraction	2 (0.6)
Atrial fibrillation	2 (0.6)
Supraventricular tachycardia	2 (0.6)
Premature ventricular contraction	6 (1.7)
Ventricular tachycardia	14 (4)
Ventricular fibrillation	2 (0.6)
Other arrhythmias (e.g., long QT interval, W wave inversion, early repolarization, poor R regression, AVD, JER, and poor QRS)	9 (2.6)
*Abnormal ECG*	290 (82.3)
**Cardiac markers**	
*Available cardiac marker^b^*	379 (97.2)
*Abnormal cardiac marker*	372 (98.4)
**Echocardiography/angiography**	
*Available echocardiography/angiography^c^*	326 (82.1)
Hypokinesia	116 (35.6)
Pericardial effusion	33 (10)
LVEF < 55%	113 (34.7)
*Abnormal echocardiography/angiography*	113 (34.7)
**CMR**	
*Available CMR^d^*	286 (75.9)
LGE/fibrosis/scar	242 (84.6)
Myocardial edema	126 (44.1)
EGE/hyperemia	12 (4.2)
Pericardial involvement	21 (7.3)
*Abnormal CMR*	259 (90)
**Histopathology examination**	
*Available histopathology examination^e^*	63 (16)
Lymphocytic	17 (27)
Lymphohistyocytic	12 (19)
Giant cell	4 (6.4)
Eosinophilic	3 (4.8)
Mixed	3 (3.8)
Neutrophil predominance: histiocyte/lymphocyte	2 (3.2)
Histiocyte predominance	1 (1.6)
Not mentioned	15 (23.8)
*Abnormal histopathology examination*	56 (89)

*Diffuse ST-segment elevation is the most common type and is observed in 71 reported cases (36% of the total ST-segment elevation). ^a^Available ECG refers to the number of cases that reported ECG examination. Its percentage is derived from the number of cases divided by the total cases (396 cases). The number of cases serves as the denominator for ECG finding/abnormality; ^b^Available cardiac marker refers to the number of cases that reported cardiac marker examination. Its percentage is derived from the number of cases divided by the total cases (396 cases). The number of cases serves as the denominator for cardiac marker abnormality; ^c^Available echocardiography/angiography refers to the number of cases that reported echocardiography/angiography examination. Its percentage is derived from the number of cases divided by the total cases (396 cases). The number of cases serves as the denominator for echocardiography/angiography findings/abnormality; ^d^Available CMR refers to the number of cases that reported CMR examination. Its percentage is derived from the number of cases divided by the total cases (396 cases). The number of cases serves as the denominator for CMR findings/abnormality; ^e^Available CMR refers to the number of cases that reported CMR examination. Its percentage is derived from the number of cases divided by the total cases (396 cases). The number of cases serves as the denominator for CMR findings/abnormality; ^f^Available histopathology examination refers to the number of cases that reported histopathology examination. Its percentage is derived from the number of cases divided by the total cases (396 cases). The number of cases serves as the denominator for histopathology examination findings/abnormality. BBB: Bundle branch block; AVD: Atrioventricular dissociation; JER: Junctional escape rhythm; CMR: Cardiac magnetic resonance; LGE: Late gadolinium enhancement; EGE: Early gadolinium enhancement.

### Cardiac markers

Eight cardiac markers were reported in this meta-summary (e.g., troponin I, hsTnI, troponin T, hsTnT, CPK, CKMB, BNP, and pro-BNP) (Table 1). Among abnormal cardiac markers, the three most commonly reported markers were troponin I (165 cases), troponin T (67 cases), and CPK (58 cases). All troponins increased in more than 90% of reported cases, whereas CPK and CKMB increased in 85.3% and 84.2% of reported cases, respectively. Moreover, hsTnT was only reported in 43 cases; however, all of those cases increased. Of 379 reported cardiac markers, 372 (98.4%) showed abnormal results.

### Radiology examination

Echocardiography or angiography information was available in 326 of 396 cases, of which 113 (34.7%) revealed abnormal results (Table 2). The three most common abnormalities were hypokinesia (35.6%), reduced left ventricular ejection fraction (LVEF) (34.7%), and pericardial effusion (10%). The median LVEF was 55%.

Of 286 CMR examinations, 259 (90%) revealed abnormal results. Based on ESC and CDC recommendations for CMR in myocarditis, the prevalence of fibrosis/scar (late gadolinium enhancement [LGE]), myocardial edema (increased global or regional myocardial signal intensity), and hyperemia (early gadolinium enhancement) in this meta-summary were 84.6%, 44.1%, and 4.2%, respectively. LGE was the most common typical myocarditis sign on CMR examination. Pericardial involvement was observed in 7.3% of CMR examinations.

### Histopathology examination

Histopathology examinations were reported in 63 (16%) cases, of which 88% revealed abnormal results. Lymphocytic and lymphohistiocytic myocarditis were the two most common histopathology subtypes, accounting for 17 (27%) and 12 (19%) examinations, respectively (Table S3). Fulminant myocarditis with extensive cardiomyolysis or necrosis was observed in 13 (20%) cases, whereas the rest of fulminant myocarditis had relatively minimal inflammation. One case had normal EMB; however, it became fulminant myocarditis during autopsy [70].

**Table 3:** Bivariate and multivariate analyses of patients characteristics and myocarditis following the first-dose vaccination

*Bivariate Analysis*				
**Variables**	**Total**	**First dose**	**Second/third doses**	***p*-value**
Age (years)^a^	369	28 (12–80)	21 (12–88)	0.00
Sex^b^				
Male	335 (87.5%)	68 (77.3%)	267 (90.5%)	0.00
Female	48 (12.5%)	20 (22.7%)	28 (9.5%)	
Comorbid factors^b^				
Presence	91 (40.8%)	19 (31.1%)	72 (44.4%)	0.10
Absence	132 (59.2%)	42 (68.9%)	90 (55.6%)	
Previous COVID-19 infection^b^				
Yes	33 (20.4%)	18 (43.9%)	15 (12.4%)	0.00
No	129 (79.6%)	23 (56.1%)	106 (87.6%)	
*Multivariate Analysis*				
**Variables**	***p*-value**	**Odds ratio**	**Lower 95% CI**	**Upper 95% CI**
Age	0.95	1.0	0.97	1.03
Sex				
Male	0.03	0.18	0.04	0.82
Female		Reference		
Previous COVID-19 infection				
Yes	0.00	5.74	2.42	13.64
No		Reference		

^a^Mann–Whitney test; ^b^Fisher’s exact test. COVID-19: Coronavirus disease 2019; CI: Confidence interval.

### Diagnosis and management

According to the CDC classification of myocarditis, 312 and 84 cases were classified as confirmed and probable groups, respectively (Table S2). Based on the ESC score for myocarditis, an ESC score of 3 (39.7%) was the most common, followed by 2 (29.5 %), 4 (18.4%), 1 (8.6%), and 5 (3.8%). The confirmed group had a significantly higher ESC score than the probable group (*p* ═ 0.00).

No specific intervention and/or observation were reported in 33 (11.8%) cases. The majority of patients were treated with nonsteroidal anti-inflammatory drugs (NSAIDs) (55%), colchicine (25%), and steroids (20%). Intravenous immunoglobulins (IVIGs) were administered in 34 (10.6%) cases. Common cardiovascular drugs were beta-blockers (16.8%), ACEis/ARBs (15.5%), and diuretics (6%). Thirty-nine (12%) cases were admitted to the intensive care unit (ICU), whereas extracorporeal membrane oxygenation (ECMO), mechanical hemodynamic support, and cardiopulmonary resuscitation (CPR) were provided to 10, 10, and 4 cases, respectively. Antiarrhythmic drugs, antiplatelets, and anticoagulants were also used in a small number of cases (Table 1). The probable group used NSAIDs more than the confirmed group (*p* ═ 0.04).

### Outcomes

Seven cases (<2%) were deceased (4 females and 3 males, respectively), whereas the other 98% recovered. BNT162b2 (4 cases) [42, 53, 125, 145] was the most common vaccine type in deceased cases, followed by Ad26.COV2.S (2 cases) [106, 113] and mRNA-1273 (1 case) [70]. Four of the seven deceased cases were older than the age of 55. All deaths occurred following the first dose of vaccination except for one case [145]. The LVEF was <30% in all deceased cases except for one case [125]. All cases had no significant comorbidity except for one case [106]. All the available data showed that the patients went to the ED on the same day of symptom onset (chest pain, 2 cases [42, 53]; hypotension, 1 case [145]; shortness of breath, 1 case; fever, 1 case [125]; palpitation, 1 case [113]; and cardiac arrest, 1 case [70]). Five of the seven cases that reported histopathology examination revealed abnormal findings (including one case that revealed normal findings in endomyocardial biopsy although revealed fulminant myocarditis in autopsy) [70]. Three of the five cases had fulminant appearance myocarditis, whereas extensive inflammation or necrotic eosinophil was noted in all cases [42, 53, 70, 106, 113, 125, 145].

### Bivariate and multivariate analyses of factors affecting myocarditis following the first-dose vaccination compared with second/third vaccinations

We analyzed demographic and comorbidity associated with myocarditis following the first dose of COVID-19 vaccination compared with the second and third doses of vaccination. Bivariate analysis showed that older age (*p* < 0.01), female sex (*p* < 0.01), and previous COVID-19 infection (*p* < 0.001) were significantly associated with the risk of myocarditis following the first dose of vaccination (Table 3). After adjusting with other variables in multivariate analysis, compared with the second and third doses of vaccination, previous COVID-19 infection was positively associated (*p* < 0.01; odds ratio [OR], 5.74; 95% confidence interval [CI], 2.42–13.64) whereas being male was negatively associated (*p* ═ 0.03; OR, 0.16; 95% CI, 0.04–0.82) with the risk of myocarditis following the first dose of vaccination (Table 3).

### Correlation analysis

The median LoH was 5 (0–79) days. The confirmed group had a significantly longer LoH than the probable group (*p* < 0.01; median, minimum–maximum: 5, 0–79 vs 3, 1–16 days). Spearman correlation showed that the LoH was positively correlated with age (*p* < 0.001; *r* ═ 0.277) and cumulative ESC score (*p* < 0.001; *r* ═ 0.251) (Table 4), whereas it was negatively correlated with LVEF (*p* < 0.001; *r* ═ −0.351).

## Discussion

This is a meta-summary of myocarditis following COVID-19 vaccination from current literature. Of note, this is not the first systematic review of the relevant topic. However, most published articles mainly investigated demographic characteristics or were limited to abstract conferences. This pooled 396 IPD provides more data for analysis (Table 1).

Hyperimmunity or immune-mediated process is suspected as one of the principal mechanisms of myocarditis following vaccination [181] through the activation of aberrant immune responses to produce severe acute respiratory syndrome coronavirus 2 (SARS-CoV-2) spike antibodies that cross-react with structurally similar cardiac-self antigens, including α-myosin heavy chain, actin [181], or even myocardial cell-expressed spike protein [151]. The nature of the higher antigenicity of mRNA vaccine in producing spike antibodies than that of other vaccine types [182] may explain our finding with higher myocarditis incidence following COVID-19 mRNA vaccination than that of other observed vaccine forms (Table 1).

**Table 4:** Spearman correlation of the length of hospitalization with LVEF, ESR, CRP, WBC, age, and ESC score

	**Age**	**LVEF**	**ESC**
	*r* ═ 0.28	*r* ═ −0.351	*r* ═ 0.251
LoH	*p* < 0.01	*p* < 0.001	*p* < 0.001
	*n* ═ 234	*n* ═ 146	*n* ═ 234

The ESC score refers to the total typical finding according to the ESC guidelines. LoH: Length of hospitalization; LVEF: Left ventricular ejection fraction; ESR: Erythrocyte sedimentation rate; CRP: C-reactive protein; WBC: White blood cell; ESC: European Society of Cardiology.

The studies included in this systematic review report cases from different populations worldwide, suggesting that the risk of myocarditis following COVID-19 vaccination is not population specific. Since we noted a higher incidence of this myocarditis in male than that in female patients (Table 1), there may be influences of hormonal differences between sex groups as the previous study explains the roles of testosterone that promotes a more aggressive immune response by inducing CD4+ cells and inhibiting anti-inflammatory immune cells, whereas estrogen suppresses pro-inflammatory lymphocytes [183]. The slightly higher proportion of female patients who developed myocarditis following the first vaccine shot than that of male patients may be because of a marginal effect (Table 3). Moreover, we reported that the median age was 22 years (Table 1). The fact that myocarditis following COVID-19 vaccination had a high incidence in the young population may also indicate the theory of hyperimmune mechanism; however, further research is needed to confirm this. Younger populations were observed to have more potent immune responses than older populations [184].

Moreover, young age was a valuable finding for diagnosing myocarditis following COVID-19 vaccination as cardiovascular disease is commonly associated with older age [12], whereas more than 60% of cases were below the age of 30. Previous COVID-19 infection may sensitize the immune system, producing a stronger immune response, even on the first vaccination dose. Our study observed that previous COVID-19 infection possessed a six-fold odd of having myocarditis following the first COVID-19 vaccine compared with the group without a previous history of COVID-19 infection (Table 3). A previous meta-analysis reported that Takotsubo cardiomyopathy, which may have similar symptoms to myocarditis, is more prevalent in female patients following the first dose of vaccination than that in young male patients following the second dose of COVID-19 vaccination [185].

The results in Table 1 indicate that comorbidities are present in less than half the number of cases. The most common comorbidity is autoimmune or inflammatory disorders, including asthma, hypothyroidism, and vitiligo. They constitute one-third of cases with comorbidities. One of the possible explanations is the aberrant immune response of patients with autoimmune-related diseases contributes to myocarditis [186]. Surprisingly, psychiatric or functional disorders, particularly attention deficit hyperactivity disorder (ADHD) and depression, constitute the second (14%) most common comorbidities, which may be related to current evidence that depression [187, 188] and ADHD [189, 190] are associated with autoimmune disorders.

Moreover, patients with a history of myocarditis constitute 11% of comorbid cases. Although those numbers were slightly smaller than the number of hypertensive cases, they could be considered high as hypertension prevalence was higher than that of myocarditis. Immune-response sensitization may contribute to recurrent myocarditis [186]. None of the studies reported myocarditis following COVID-19 vaccination in pregnancy. Pregnancy seems neither a risk factor for developing myocarditis nor it is a protective factor. Regarding immune mediation as the basic mechanism, the immune system suppression state in pregnancy [191, 192] may diminish the aberrant immune response to COVID-19 vaccination.

The preceding symptoms of myocarditis following COVID-19 vaccination were not specific (e.g., fever, myalgia, and chill) and only occurred in approximately half of the cases (Table 1). Chest pain should be the first specific clue of myocarditis following COVID-19 vaccination diagnosis and the main reason nearly all patients (80.0%) came to the ED (Table 1). Considering that cardiovascular comorbidities are relatively high, it is mandatory to distinguish chest pain between myocarditis and other cardiovascular diseases [12]. Chest pain was commonly felt less than four days following the vaccine shot, and most patients came to the ED on the same day of specific symptom onset (Table 1). Interestingly, confirmed patients tended to seek help in the ED faster than probable patients (Table 1).

As previously stated, myocardial inflammation is the primary underlying process and is expected to be depicted by laboratory examination results. We observed higher CRP levels in 78.2% of patients, whereas WBC and ESR increments were reported in approximately 40% of patients (Table 1). Indeed, inflammatory parameters may be less sensitive and specific. Cardiac-specific markers are considered far more sensitive and increased in most cases, particularly troponins (Tables 1 and 2). Natriuretic peptides were highly sensitive to rule out heart failure [193], whereas only a few cases posed symptoms. In addition to cardiac markers, admission ECG is an inexpensive and fairly sensitive examination. ST-segment abnormality could be observed in 70% of cases, mainly consisting of diffuse ST-segment elevation (Table 3). Diffuse ST-segment elevation is a specific sign of myocarditis. However, it only presented in half of the cases. The combination of cardiac markers and ECG was a sensitive early screening modality for ruling out myocarditis following COVID-19 vaccination. However, coronary angiography may be needed to rule out acute coronary syndrome since both would result in similar findings.

Histopathology examination revealed abnormal findings in 90% of cases (Table 2). Myocardial inflammation in myocarditis has a patchy nature [194], and a negative result may be because of inadequate sample collection or sampling bias during sample fixation, processing, or preparation [140, 195]. Nonetheless, histopathology examination has an imperative standing position as the gold standard diagnosis modality, drawing possible etiology of myocarditis, and considering appropriate management [183]. Acute lymphocytic and lymphohistiocytic myocarditis are the commonest histopathology subtypes in this review (Table 2), and both of them seem to be associated with immune-mediated etiologies [195, 196]. The immune-mediated mechanism is often associated with minimal inflammation or myocytolysis [195], which was also observed in this meta-summary [54, 57, 68, 88, 118, 120, 121, 145, 148, 151]. In 9 of 15 patients, the cardiomyocytes expressed SARS-CoV-2 spike proteins [151] that may provoke cardiac tissue inflammation following COVID-19 mRNA vaccination. Although human data are limited, surprisingly, cardiomyocytes in mice also expressed spike proteins intracellularly following a vaccine shot [197]. In most cases, a shift from CD8+ T cells to CD4+ T cells was noted [151], wherein CD4+ T cell is one of the major drivers in immune-mediated myocarditis [198]. Human leukocyte antigen-DR (HLA-DR), an HLA class II region commonly observed in several immune-mediated diseases, was increasingly expressed in 11 of 14 patients following COVID-19 vaccination [151]. Additionally, perforin-mediated myocardial lysis associated with acute idiopathic [199] and viral myocarditis [200, 201] was not observed in 15 patients who received a vaccine shot [151]. As the temporal association between vaccination is strong and the absence of other alternative causes (e.g., negative viral panel), the COVID-19 vaccine is probably the primary inducer of those complex immune-mediated processes.

Furthermore, infiltrating eosinophils were noted in 24% of all histopathology reports (Table 3) and 100% of all deceased cases. Eosinophils are known as one of the effectors in hypersensitivity, and their activation promotes the secretion of their large granules to provoke inflammatory and cytotoxic effects [202], which may explain why the presence of eosinophils may be associated with higher mortality.

According to the latest ESC and CDC criteria, CMR is a significant alternative modality to EMB in myocarditis diagnosis. In this review, CMR sensitivity is similar to EMB (Table 3). CMR provides noninvasive tissue characterization, including inflammatory stages and patterns, which may be used to rule out the ischemic cause, a common etiology that mimics myocarditis [12]. In the present cohort, myocardial fibrosis or scar represented by LGE, followed by myocardial edema were the two most common typical findings to confirm the diagnosis of myocarditis following COVID-19 vaccination. Diagnosis should not rely on pleural involvement as it is not a specific sign of myocarditis and is only positive in 10% of cases.

In a recent study, the vast majority of patients were treated with NSAIDs, thereby accelerating the resolution of myocardial inflammation [203] (Table 1). However, its position remains inconclusive in myocarditis [12]. IVIGs were commonly used for ICU patients to modulate the immune system and inflammatory response [204], which is proposed as the basic pathophysiology of myocarditis following COVID-19 vaccination [183]. Colchicine seems promising in treating pericarditis [205] and decreasing inflammation in myocarditis [206]. Notwithstanding, more clinical trials are needed regarding the finding of its toxicity in a pre-clinical study [207]. The American Heart Association considers steroids in severe cases although steroid position was unclear as most cases of myocarditis following COVID-19 vaccination were mild to moderate. In summary, the chief management of myocarditis remains supportive, and specific guideline-based therapy was directed for common complications, such as heart failure and arrhythmia [12]. Nevertheless, to conclude the optimal management for myocarditis following mRNA vaccination, further research is needed.

The confirmed group had a longer LoH than the probable group (Table 1). One of the possible explanations was that patients with CMR and EMB findings may be treated more carefully and appropriately or they tended to have severe clinical manifestations; therefore, they have longer LoH. Interestingly, LoH is correlated with cumulative ESC scores (Table 4). Patients with longer LoH tended to have older age and lower LVEF (Table 4). Generally, older age and lower LVEF are associated with more severe outcomes [208, 209], including myocarditis following COVID-19 vaccination.

Histopathology examination revealed that myocarditis following COVID-19 vaccination is associated with a relatively good prognosis. Dominant lymphocyte infiltration cells as the most common subtype observed in this meta-summary is associated with better outcomes than other subtypes [194], especially since the majority of cases showed minimal inflammation and cardiomyolysis. Moreover, negative perforin cells observed in myocarditis following COVID-19 vaccination were associated with better LVEF improvement [210] as in this review. These may explain why most myocarditis following COVID-19 vaccination had relatively benign characteristics.

Deceased cases are minor (<2%) and seem to have distinct characteristics (Table S2). Compared with overall cases, deceased cases seem likely to occur in female patients (67% vs 12%), those without previous COVID-19 infection who present following the first dose of vaccine shot (86% vs 23%), and in older age (70% death in patients aged >55 years). The geriatric population seems to have a higher mortality rate due to myocarditis since this may be because of the senescence physiologic state in the geriatric population during compensated hemodynamic collapses and other pathological conditions. Notably, all geriatric deaths had an LVEF of <30% (Table S2). Owing to unspecific symptoms, underdiagnosis may also occur and increase mortality [42]. Therefore, early warning and identification are needed.

We observed that the deceased cases have sudden and severe clinical manifestations, which may be because of the following reasons: (1) all of them went to the ED on the day of specific onset, (2) severely reduced LVEF (<30%) in 4 of 5 cases, (3) extensive myocardial damage in the majority of the cases, (4) progressive histopathology examination from normal to fulminant myocarditis, and (5) rapidly progressed disease to death in less than 2 days after admission in more than half of the cases. Several cases have been treated with heart failure drugs (Table 1). As the majority of EMB following COVID-19 vaccination showed non-infectious myocarditis and the presence of eosinophil infiltrates in all deceased cases, early immunosuppressant drugs may be required in addition to heart failure and intensive therapy [12], particularly in fulminant myocarditis. However, EMB remains the gold standard for diagnosing and determining etiology and appropriate therapy in myocarditis [12, 195].

The limitation of this meta-summary was that it consisted of case reports and case series, which are the lowest levels in the hierarchy of evidence and may lack reported data. However, Sampayo-Cordero et al. [211] reported that aggregating case reports or case series and quantitatively analyzing them showed similar results to the meta-analysis of clinical study. A meta-summary of case reports was helpful in the setting of scarce data [212, 213]. We anticipated the bias by assessing the included articles. In the present meta-summary, no confirmed cases were diagnosed by histopathology examination; however, CMR was a recommended [13] and valuable noninvasive modality to confirm myocarditis following COVID-19 vaccination.

This is not the first review of the relevant topic. Overall, the results of this systematic review were concordant with those of published reviews. For example, myocarditis following vaccination has a preponderance of young adolescents and males; occurs following the second dose of mRNA vaccine shot; and is characteristically manifested by chest pain, elevated cardiac markers, and benign outcomes [214–218]. However, almost all of the included articles did not gather the specific IPD and focused on the pooled prevalence of each variable. In this systematic review, we collected the IPD of each case. Therefore, this meta-summary of cases allowed for statistical analysis to evaluate the comparison (e.g., comparison between probable and definitive cases), correlation (e.g., the correlation between LVEF and LoH to potential prognostic variables), and association (e.g., multivariate analysis of cases with previous COVID-19 infection had a six-fold odds of myocarditis following the first dose of vaccine shot than those without COVID-19 history) that were scarcely observed in previous reviews. Furthermore, this systematic review quantitatively analyzed more variables, including from the day of specific pain to the ED admission and prodromal symptoms, which were not reported in other reviews. Lastly, the detailed variable description (Table S2 and S3), including 63 histopathology examinations, was one of the strengths of this systematic review.

## Conclusion

Patients complaining of chest pain, particularly young males, following the second dose of mRNA COVID-19 vaccination should be suspected of myocarditis. Cases of myocarditis following the first vaccination dose are associated with previous COVID-19 infection and being female. Comorbidities are present in half of the cases, and half of them are dysregulated immune-associated diseases. More attention should be provided to older females who come to the ED following the first dose of COVID-19 vaccination, even when their comorbidities are not significant and they present non-chest pain symptoms, as those characteristics dominate most deceased cases. The combination of ECG and cardiac markers, particularly troponins, is a highly sensitive screening modality, and their normal values may be used to rule out myocarditis, whereas CMR is preferred as a non-invasive examination to confirm myocarditis. Histopathology examination has an imperative value as the gold standard modality to diagnose, determine possible etiology, and guide appropriate management, particularly in severe and confusing cases. Non-infective subtypes dominate histopathology examinations with eosinophil involvement. Histopathology and epidemiology evidence indicate that immune-mediated process is the underlying mechanism of myocarditis following COVID-19 vaccination. The management is mainly supportive.

## Supplemental Data

**Table S1:** Critical appraisal of included studies. Critical appraisal table of included case reports according to the Joanna Briggs Institute Critical Appraisal Tools 2017

**No**	**Study**	**Q1**	**Q2**	**Q3**	**Q4**	**Q5**	**Q6**	**Q7**	**Total (Yes)**	**Total (%)**	**Category**
1	Adzaki et al. 2021	Unclear	Yes	Yes	No	Yes	Yes	Yes	5	71.43	Medium
2	Agdamag et al. 2022	Yes	Yes	Yes	Yes	Yes	Yes	Yes	7	100.00	Low
3	Albert et al. 2021	Unclear	Unclear	Yes	Yes	Yes	Yes	Yes	5	71.43	Medium
4	Alizadeh et al. 2022	Unclear	Yes	Yes	Yes	Yes	Yes	Yes	6	85.71	Low
5	Ameratunga et al. 2022	Unclear	Yes	Yes	Yes	Yes	Yes	Yes	6	85.71	Low
6	Ammirati et al. 2021	Unclear	Yes	Yes	Yes	No	No	Yes	4	57.14	Medium
7	Ansari et al. 2022	Unclear	Yes	Yes	Yes	Yes	Yes	Yes	6	85.71	Low
8	Asaduzzaman et al. 2022	Unclear	Yes	Yes	Yes	No	No	Yes	4	57.14	Medium
9	Azir et al. 2021	Unclear	Yes	Yes	Yes	Unclear	No	Yes	4	57.14	Medium
10	Bae et al. 2022	Unclear	Yes	Yes	Yes	Yes	Yes	Yes	6	85.71	Low
11	Banala et al. 2022	Unclear	Yes	Yes	Yes	Yes	Yes	Yes	6	85.71	Low
12	Bartlett et al. 2022	Unclear	Yes	Yes	Yes	Yes	Yes	Yes	6	85.71	Low
13	Bitar et al. 2022	Unclear	Yes	Unclear	Yes	Yes	Yes	Yes	5	71.43	Medium
14	Brage et al. 2022	Yes	Yes	Yes	Yes	Yes	Yes	Yes	7	100.00	Low
15	Bucur et al. 2022	Yes	Yes	Yes	Yes	No	Yes	Yes	6	85.71	Low
16	Cereda 2021	Unclear	Yes	No	Yes	Yes	Yes	Yes	5	71.43	Medium
17	Chacar et al. 2021	Yes	Yes	No	Yes	Yes	Yes	Yes	6	85.71	Low
18	Chellapandian et al. 2021	Yes	Yes	Yes	Yes	Yes	Yes	Yes	7	100.00	Low
19	Choi et al. 2021	Yes	Yes	Yes	Yes	Yes	Yes	Yes	7	100.00	Low
20	Chow et al. 2022	Unclear	Yes	Yes	Yes	Yes	Yes	Yes	6	85.71	Low
21	Cimaglia 2021	Unclear	Yes	Yes	Yes	No	Yes	Yes	5	71.43	Medium
22	D’angelo et al. 2021	Unclear	Yes	No	Yes	Yes	Yes	Yes	5	71.43	Medium
23	Das et al. 2022	Yes	Yes	Yes	Yes	Yes	Yes	Yes	7	100.00	Low
24	Deb et al. 2021	Unclear	Yes	Yes	No	Yes	Yes	Yes	5	71.43	Medium
25	Dlewati et al. 2022	Unclear	Yes	Yes	No	Yes	Yes	Yes	5	71.43	Medium
26	Ehrlich et al. 2021	Unclear	Yes	Yes	Yes	Unclear	Unclear	Yes	4	57.14	Medium
27	Elhouderi 2022	Yes	No	Yes	No	Yes	Yes	Yes	5	71.43	Medium
28	Etuk et al. 2022	Unclear	Yes	Yes	Yes	Yes	Yes	Yes	6	85.71	Low
29	Fadah et al. 2022	Unclear	Yes	Yes	Yes	Yes	Yes	Yes	6	85.71	Low
30	Farooq et al. 2022	Unclear	No	Yes	Yes	Yes	Yes	Yes	5	71.43	Medium
31	Fritz et al. 2022	Unclear	Yes	Yes	Yes	Yes	Yes	Yes	6	85.71	Low
32	Garcia et al. 2021	Unclear	Yes	Yes	Yes	Unclear	Yes	Yes	5	71.43	Medium
33	Gautam et al. 2021	Yes	Yes	Yes	Yes	No	No	Yes	5	71.43	Medium
34	Generette et al. 2022	Unclear	Yes	Yes	Yes	Yes	Yes	Yes	6	85.71	Low
35	Gill Jashan 2022	Unclear	Yes	Yes	Yes	Yes	Yes	Yes	6	85.71	Low
36	Habedank et al. 2021	Unclear	Yes	Yes	Yes	No	Yes	Yes	5	71.43	Medium
37	Habib 2021	Yes	Yes	Yes	Yes	Yes	Yes	Yes	7	100.00	Low
38	Hasnie et al. 2021	Unclear	Yes	Yes	Yes	Yes	Yes	Yes	6	85.71	Low
39	Hassanzadeh et al. 2022	Yes	Yes	Yes	No	Yes	Yes	Yes	6	85.71	Low
40	Hirsch et al. 2022	Yes	Yes	Yes	Yes	Yes	Yes	Yes	7	100.00	Low
41	Horiuchi et al. 2022	Unclear	Yes	No	Yes	No	Yes	Yes	4	57.14	Medium
42	Hoshino et al. 2022	Unclear	Yes	Yes	Yes	Yes	Yes	Yes	6	85.71	Low
43	Hung et al. 2021	Unclear	Yes	Yes	Yes	Yes	Yes	Yes	6	85.71	Low
44	Isaak et al. 2021	Unclear	Yes	No	Yes	No	Yes	Yes	4	57.14	Medium
45	Iwamuro et al. 2022	Unclear	Yes	No	Yes	Yes	Yes	Yes	5	71.43	Medium
46	Jamal et al. 2022	Unclear	Yes	Yes	Yes	Yes	Yes	Yes	6	85.71	Low
47	Kadwalwala et al. 2021	Unclear	Yes	Yes	Yes	Yes	Yes	Yes	6	85.71	Low
48	Kang et al. 2022	Unclear	Yes	No	Yes	Yes	Yes	Yes	5	71.43	Medium
49	Kawakami et al. 2022	Unclear	Yes	Unclear	Yes	Yes	Yes	Yes	5	71.43	Medium
50	Kawano et al. 2022	Yes	Yes	Yes	Yes	Yes	Yes	Yes	7	100.00	Low
51	Kawauchi et al. 2022	Unclear	Yes	Yes	Yes	Unclear	Yes	Yes	5	71.43	Medium
52	Kelle et al. 2022	Yes	Unclear	Yes	Yes	Unclear	Unclear	Yes	4	57.14	Medium
53	Kim Cheol et al. 2021	Unclear	Yes	Yes	Yes	Unclear	Yes	Yes	5	71.43	Medium
54	Kim Dongwon et al. 2021	Yes	Yes	Unclear	Yes	Yes	Yes	Yes	6	85.71	Low
55	Kim Hyun et al. 2022	Unclear	Yes	Yes	Yes	Yes	Yes	Yes	6	85.71	Low
56	Kim Hyung et al. 2022	Unclear	Yes	Yes	Yes	Yes	Yes	Yes	6	85.71	Low
57	Kimball et al. 2022	Unclear	Yes	Yes	Yes	Yes	Yes	Yes	6	85.71	Low
58	Kojima et al. 2022	Yes	Yes	Unclear	Yes	Yes	Yes	Yes	6	85.71	Low
59	Korsoglou et al. 2022	Unclear	Yes	Yes	Yes	Yes	Yes	Yes	6	85.71	Low
60	Kyaw et al. 2022	Unclear	Yes	Yes	No	Yes	Yes	Yes	5	71.43	Medium
61	Lim et al. 2021	Unclear	Yes	No	Yes	No	Yes	Yes	4	57.14	Medium
62	Lin et al. 2022	Yes	Yes	Yes	No	Yes	Yes	Yes	6	85.71	Low
63	Loch et al. 2022	Unclear	Yes	Yes	Yes	Yes	Yes	Yes	6	85.71	Low
64	Mangesha et al. 2022	Yes	Yes	Yes	Yes	Yes	Yes	Yes	7	100.00	Low
65	Marsukjai et al. 20222	Yes	Yes	Yes	Yes	Unclear	Yes	Yes	6	85.71	Low
66	McCullough et al. 2021	Yes	Yes	Yes	Unclear	Yes	Yes	Yes	6	85.71	Low
67	McLean 2021	Unclear	Yes	Yes	Yes	Yes	Yes	Yes	6	85.71	Low
68	Mimouni et al. 200	Unclear	Yes	Yes	Yes	Yes	Yes	Yes	6	85.71	Low
69	Minocha et al. 2021	Unclear	Yes	Yes	Yes	Unclear	Yes	Yes	5	71.43	Medium
70	Miqdad et al. 2022	Unclear	Unclear	Yes	Unclear	Yes	Yes	Yes	4	57.14	Medium
71	Mohammed et al. 201	Yes	Yes	Yes	Yes	Yes	Yes	Yes	7	100.00	Low
72	Morton et al. 2022	Yes	Unclear	Yes	Yes	Yes	Yes	Yes	6	85.71	Low
73	Murakami et al. 2022	Yes	Unclear	Yes	Yes	Yes	Yes	Yes	6	85.71	Low
74	Murase et al. 2022	Yes	Unclear	Yes	Yes	Yes	Unclear	Yes	5	71.43	Medium
75	Muthukumar et al. 2021	Unclear	Yes	Yes	Yes	Yes	Yes	Yes	6	85.71	Low
76	Nagasaka et al. 2022	Yes	Yes	Yes	Yes	Yes	Yes	Yes	7	100.00	Low
77	Naghashzadeh et al. 2022	Yes	Yes	Yes	Yes	Yes	Yes	Yes	7	100.00	Low
78	Nassar et al. 2021	Yes	Yes	Unclear	No	Yes	Yes	Yes	5	71.43	Medium
79	Nguyen et al. 2021	Yes	Yes	Yes	Unclear	Unclear	Unclear	Yes	4	57.14	Medium
80	Nitish et al. 2022	Yes	Yes	Yes	Yes	Yes	Yes	Yes	7	100.00	Low
81	Nunn et al. 2022	Yes	Yes	Yes	Yes	Unclear	Unclear	Yes	5	71.43	Medium
82	Oh etal al. 2022	Yes	Yes	Yes	Yes	Yes	Yes	Yes	7	100.00	Low
83	Ohnishi et al. 2022	Unclear	Yes	Yes	Yes	Yes	Yes	Yes	6	85.71	Low
84	Ohtani et al. 2022	Yes	Yes	Yes	Yes	Yes	Yes	Yes	7	100.00	Low
85	Oka et al. 2022	Yes	Yes	Yes	Yes	Yes	Yes	Yes	7	100.00	Low
86	Olmos et al. 2022	Yes	Yes	Yes	Yes	Yes	Yes	Yes	7	100.00	
87	Olagunju et al. 2022	Yes	Yes	Yes	Unclear	Yes	Unclear	Yes	5	71.43	Medium
88	Onderko et al. 2021	Yes	Yes	Yes	Yes	Unclear	Yes	Yes	6	85.71	Low
89	Pantsios et al. 2022	Yes	Yes	Yes	Yes	Unclear	Yes	Yes	6	85.71	Low
90	Park and You 2022	Yes	Yes	Yes	Unclear	Yes	Unclear	Yes	5	71.43	Medium
91	Pasha et al. 2022	Yes	Yes	Yes	Yes	Yes	Unclear	Yes	6	85.71	Low
92	Patel et al. 2022	Unclear	Unclear	Yes	Yes	Yes	Yes	Yes	5	71.43	Medium
93	Patrignani et al. 2021	Unclear	Yes	Yes	Yes	Unclear	Yes	Yes	5	71.43	Medium
94	Rasbi et al. 2022	Unclear	Yes	Yes	Unclear	Yes	Yes	Yes	5	71.43	Medium
95	Riddell et al. 2022	Unclear	Yes	Yes	Yes	Yes	Yes	Yes	6	85.71	Low
96	Sano et al. 2022	Yes	Yes	Yes	Yes	Unclear	Yes	Yes	6	85.71	Low
97	Satomi et al. 2022	Yes	Yes	Yes	Yes	Yes	Yes	Yes	7	100.00	Low
98	Schmitt et al. 2021	Yes	Yes	Yes	Yes	Yes	Yes	Yes	7	100.00	Low
99	Sciaccaluga et al. 2022	Yes	Unclear	Yes	Yes	Yes	Yes	Yes	6	85.71	Low
100	Sharbatdaran et al. 2022	Yes	Yes	Yes	Yes	Yes	Yes	Yes	7	100.00	Low
101	Shumkova et al. 2021	Unclear	Yes	Yes	Yes	Yes	Yes	Yes	6	85.71	Low
102	Singh et al. 2021	Unclear	Yes	Yes	Yes	No	Yes	Yes	5	71.43	Medium
103	Sokolska et al. 2021	Unclear	Yes	Yes	Yes	No	No	Yes	4	57.14	Medium
104	Takase et al. 2021	Yes	Unclear	Yes	Yes	Yes	Yes	Yes	6	85.71	Low
105	Tinoco et al. 2021	Unclear	Yes	Yes	Yes	Yes	Yes	Yes	6	85.71	Low
106	Tomohiko et al. 2022	Yes	Yes	Yes	Yes	Yes	Yes	Yes	7	100.00	Low
107	Torres et al. 2021	Unclear	Yes	Yes	No	Yes	Yes	Yes	5	71.43	Medium
108	Ture et al. 2022	Unclear	Yes	Yes	No	Yes	Yes	Yes	5	71.43	Medium
109	Uesako et al. 2022	Yes	Yes	Yes	Yes	Yes	Yes	Yes	7	100.00	Low
110	Ujueta et al. 2021	Yes	Yes	Yes	Yes	Yes	Yes	Yes	7	100.00	Low
111	Watanabe et al. 2022	Yes	Yes	Yes	Yes	Yes	Yes	Yes	7	100.00	Low
112	Watkins et al. 2021	Unclear	Yes	Yes	Unclear	Yes	Yes	Yes	5	71.43	Medium
113	Williams et al. 2021	Unclear	Unclear	Yes	Yes	Yes	Yes	Yes	5	71.43	Medium
114	Wong et al. 2022	Yes	Unclear	Yes	Yes	Yes	Yes	Yes	6	85.71	Low
115	Wu Bryan et al. 2022	Unclear	Yes	Yes	Yes	Yes	Yes	Yes	6	85.71	Low
116	Wu Chia et al. 2022	Yes	Yes	Yes	Yes	Yes	Yes	Yes	7	100.00	Low
117	Yamamoto et al. 2022	Yes	Unclear	Yes	Yes	Unclear	Unclear	Yes	4	57.14	Medium
118	Yamamoto et al. 2022	Yes	Yes	Yes	Yes	Yes	Yes	Yes	7	100.00	Low
119	Yen et al. 2022	Yes	Yes	Yes	Yes	Unclear	Unclear	Yes	5	71.43	Medium
120	Zaveri et al. 2021	Yes	Yes	Yes	Yes	Unclear	Yes	Yes	6	85.71	Low
Total (%)	46.7	88.3	89.2	85.8	76.7	86.7	100.8				

Question 1 Were patient’s demographic characteristics clearly described?

Question 2 Was the patient’s history clearly described and presented as a timeline?

Question 3 Was the current clinical condition of the patient on presentation clearly described?

Question 4 Were diagnostic tests or assessment methods and the results clearly described?

Question 5 Was the intervention(s) or treatment procedure(s) clearly described?

Question 6 Was the post-intervention clinical condition clearly described?

Question 7 Does the case report provide takeaway lessons?

Result analysis

Low risk of bias 74

Medium risk of bias 45

High risk of bias 2

**Tables S2 and S3.** Owing to the numerous reported data, we provided our supplementary files 2 and 3 in the form of.xls document that could be accessed at https://drive.google.com/drive/folders/1BNINLw22ul4-cFWL2yerImPUDoQ2x77I?usp=shari.
